# Supplementary material for: Physical Activity and Low Glycemic Index Mediterranean Diet: Main and Modification Effects on NAFLD Score. Results from a Randomized Clinical Trial
Source: Nutrients. 2020 Dec 28;13(1):66. doi: 10.3390/nu13010066 (PMC7823843; doi:10.3390/nu13010066)
Supplement: Supplementary file 1 [file nutrients-13-00066-s001.zip › nutrients-1053716 - Supplementary S1 - Detailed Information about Control Diet.pdf]

progetto

NU TRI A T T

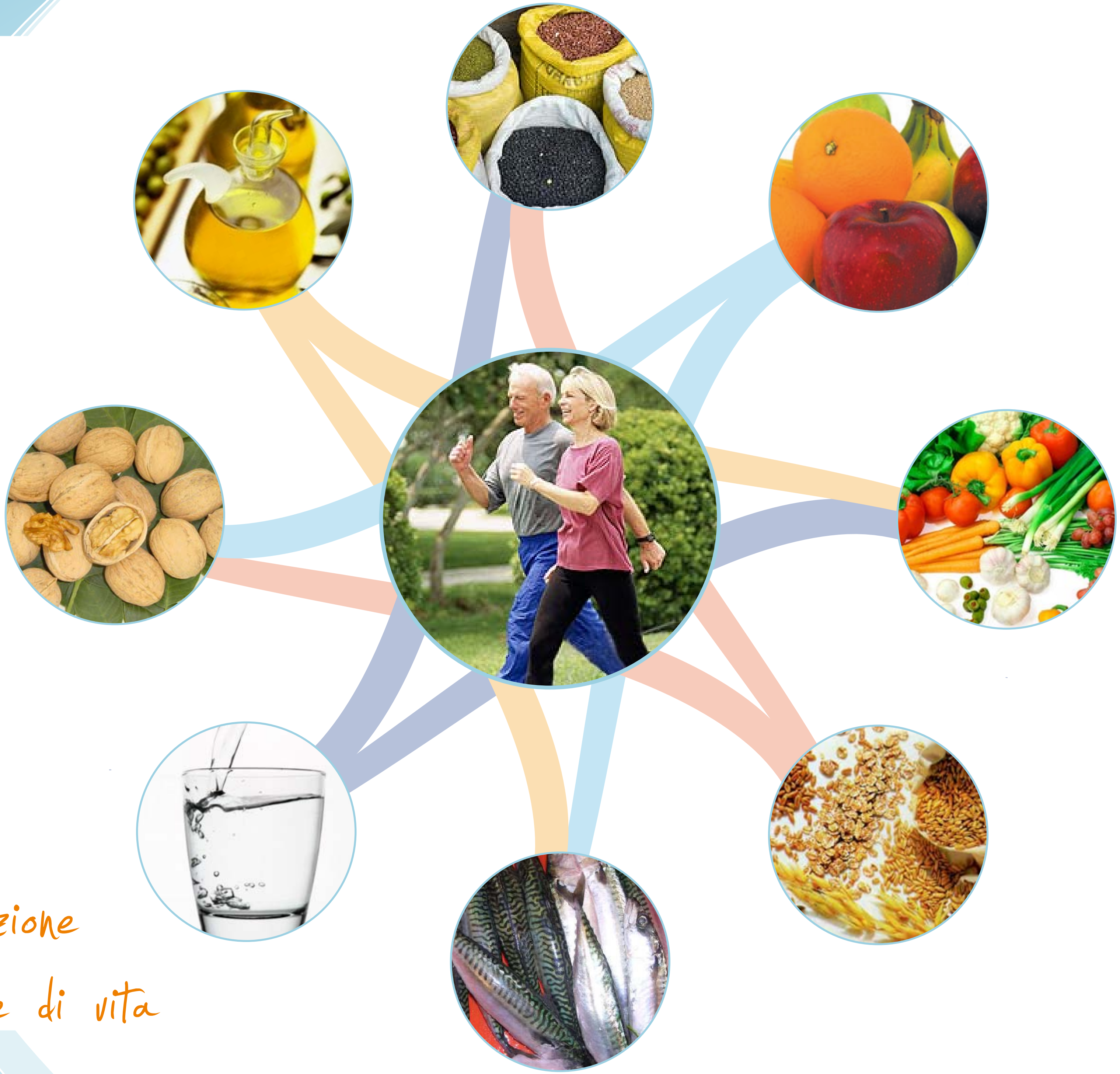

Una sana alimentazione

Un corretto stile di vita

PREMESSA

La salute è nelle tue mani, la costruisci ogni giorno quando vai al supermercato e scegli un cibo piuttosto che un altro, quando prepari un pranzo o una cena. In questi momenti fai scelte alimentari che influenzeranno profondamente la tua salute. Infatti una scorretta alimentazione determina la comparsa di una serie di fattori di rischio per le malattie cardiovascolari ed i tumori. Una delle prime manifestazioni di questi fattori di rischio è rappresentato dalla Steatosi Epatica.

L'analisi dei tuoi dati fisiologici e di laboratorio, provenienti dallo studio Nutriep cui hai partecipato, ha evidenziato che hai la Steatosi Epatica (fegato grasso) e quindi sei a maggiore rischio di sviluppare una malattia cardiovascolare o un tumore, patologie che costituiscono la principale causa di morte nel mondo occidentale.

Il Laboratorio di Epidemiologia dell 'IRCCS "S. De Bellis" di Castellana Grotte ha attivato il progetto di Ricerca: "NutriAtt: Effetto della dieta Mediterranea a basso Indice Glicemico, di due programma di attività fisica di diversa intensità e della loro interazione sulla steatosi Epatica di grado medio ed elevato".

Ti invitiamo, pertanto, a partecipare a questo studio che ci aiuterà a comprendere quale sia la dieta più efficace nel trattamento della Steatosi Epatica. Le indicazioni alimentari descritte nelle pagine seguenti sono quelle quelle dettate dall' Organizzazione Mondiale Della Sanità (OMS) , indicazioni che ti chiediamo di seguire. Pertanto ti preghiamo di registrare la tua dieta giornaliera su questo diario alimentare per tutto il periodo dello studio, per permetterci di valutare il tuo livello di aderenza alla Dieta che ti abbiamo consigliato.

ATTENTO AL SEMAFORO,
CONTROLLA LE TUE SCELTE ALIMENTARI

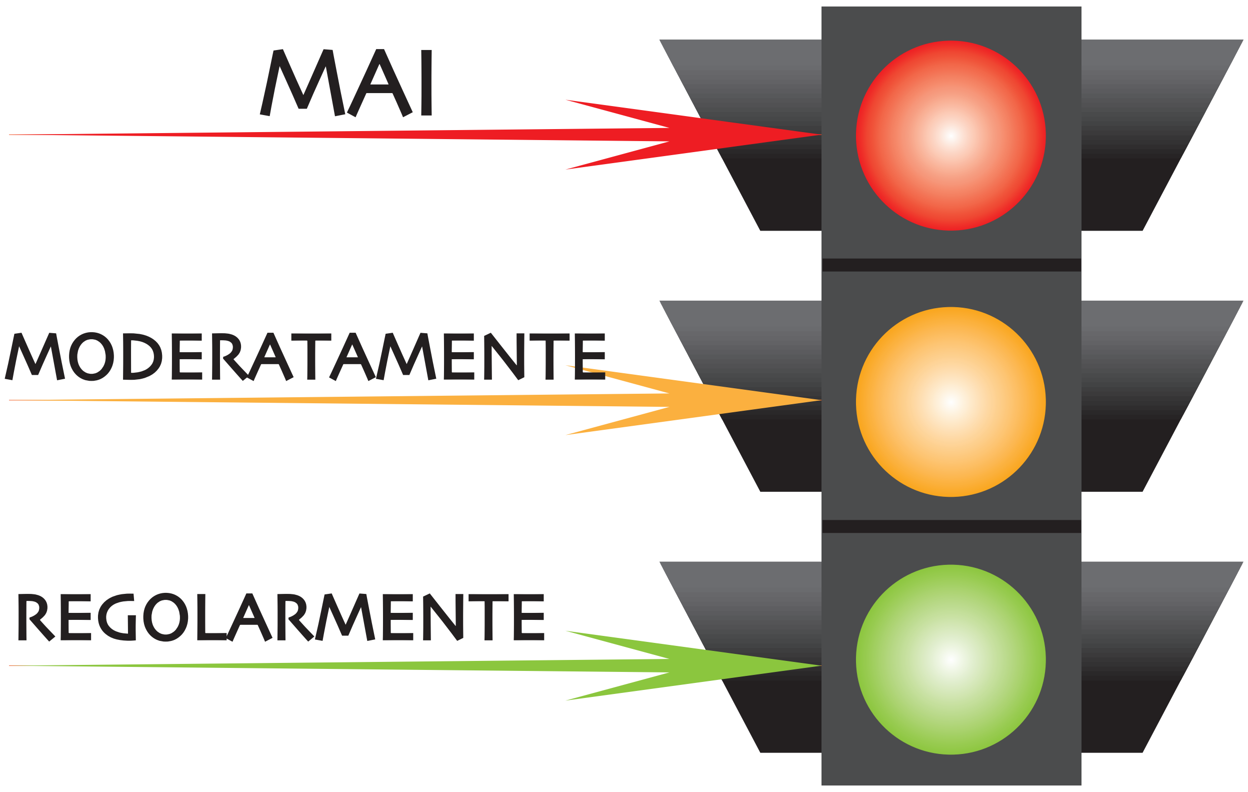

La scelta degli alimenti e la frequenza della loro assunzione è cosa fondamentale per impostare un corretto programma alimentare che, giorno per giorno, dovrebbe essere seguito da tutti per mantenere uno stato di salute soddisfacente.

Per orientare le scelte degli alimenti che meglio rappresentano le **indicazioni dell' Organizzazione Mondiale della Sanità** (OMS), abbiamo contraddistinto con il colore **VERDE** i cibi da assumere regolarmente, con il **GIALLO** quelli da consumare con moderazione e con il **ROSSO** quelli da evitare.

REGOLARMENTE

1 - VERDURA CRUDA
(insalata verde, pomodori, cetrioli, sedano carote, ravanelli, ecc.)

2 - VERDURA COTTA A VAPORE O LESSATA
(bietole, rape, cicorie, cavolfiori, broccoli, ecc.)

45 -LEGUMI SECCHI
(lenticchie, ceci, fagioli, fave, soia)

46 -LEGUMI FRESCHI
(piselli, fave, fagiolini)

10 - PASTA
16 - RISO
23 - PATATE
27 - SUGO DI POMODORO
28 - OLIO EXTRA VERGINE D'OLIVA (a crudo)
20 - PANE COMUNE
53 - CRACKERS, SALATINI, GRISSINI, TARALLI, FOCACCE E CEREALI

34 - CARNE BIANCA
(pollo, tacchino, coniglio)

39 - PESCE

3 - FRUTTA FRESCA
(mele, pere, arance, pompelmo, kiwi, ecc.)

54 - BISCOTTI SECCHI, FETTE BISCOTTATE

64 - CAFFE'

52 - ACQUA NATURALE

MODERATAMENTE

	max
30-LATTE E YOGURT	2 volte/di
32-LATTICINI (mozzarella, scamorza, ricotta) 31-FORMAGGI STAGIONATI (grana, parmigiano, pecorino, ecc.)	3 volte/sett.
33-UOVA	2 volte/ sett. (un uovo).
33-CARNE ROSSA (manzo, vitello, maiale, cavallo, ecc.)	2 volte/sett.
37-RAGU'	1 volta/sett.
38-SALUMI E INSACCATI (prosciutto crudo o cotto, bresaola, speck, salame, mortadella, ecc.	2 volte/sett.
47-PIZZA	1 volta/sett.
23-PATATE	2 volte/sett.
6-FRUTTA SECCA (noci, mandorle, datteri, fichi secchi, ecc.)	2 volte/sett.
50-BEVANDE NON ALCOLICHE GASSATE E NON (aranciata, coca-cola, chinotto, succo di frutta, ecc.)	1 volta/sett.
48-BIRRA	1 volta/sett. (max 33 cl.)
49-VINO	2 volte/di (un bicchiere)
44-TONNO IN SCATOLA 40-MOLLUSCHI E CROSTACEI	2 volte/sett.
61-MARMELLATE 59-GELATI E GRANITE	2 volte/sett.
57-TORTE, DOLCI, PASTICCINI	1 volta/sett.

MAI

51 - BEVANDE ALCOLICHE E SUPER ALCOLICHE
(brandy, grappa, liquori, whisky, spumante)

24 - PATATE FRITTE

67 - FRITTURA IN GENERE

68 - SALSICCE E PANCETTA

29 - BURRO, MARGARINA, MAIONESE, PANNA

69 - FORMAGGI MOLTO GRASSI
(burrata, mascarpone, gorgonzola, formaggio cremoso spalmabile, ecc.)

9 - FRUTTA SCIROPPATA E CANDITA

58 - CORNETTI, BRIOCHES, MERENDINE

60 - CARAMELLE E CIOCCOLATE

IL TUO DIARIO ALIMENTARE

Istruzioni per la compilazione

- E' fondamentale che il Diario venga compilato tutti i giorni dopo i pasti o appena possibile.
- Il Diario è costituito da una serie di fogli, uno per settimana. In ogni foglio settimanale, i giorni da lunedì a domenica sono suddivisi in colazione, pranzo, cena e fuori pasto.
- Registra tutti i cibi e tutte le bevande che consumi durante la giornata (compresi la domenica ed i giorni festivi).
- La registrazione consisterà nel riportare in corrispondenza del pasto il numero identificativo dell'alimento/bevanda o del gruppo di alimenti consumato che troverai nella lista che abbiamo allegato. Se non trovi nella nostra lista l'alimento che hai consumato, scrivi direttamente il suo nome nello spazio riservato al pasto corrispondente.

Esempio:

Descriviamo di seguito un ipotetico giorno del diario compilato dal Sig. Rossi.

Al termine di ciascun pasto il Sig. Rossi ha registrato ciò che ha mangiato e bevuto, per cui ha scritto nella casella del pasto specifico il numero corrispondente all'alimento consumato (consultando la lista degli alimenti).

A colazione ha bevuto una tazza di latte e caffè con 1 cucchiaino di zucchero e ha mangiato una mela.

	COLAZIONE	Fuori pasto	PRANZO	Fuori pasto	CENA	Fuori pasto	ATTIVITÀ FISICA
LUNEDÌ	30+64+63<br>3						☐ Nessuna<br>☐ Passeggiata<br>☐ Lavori domestici<br>☐ Palestra<br>☐ Altro (specificare)<br>________

A pranzo ha mangiato un piatto di lenticchie senza pasta condito con olio extra vergine d' oliva crudo, un merluzzo, un piatto di ortaggi crudi sconditi e ha bevuto due bicchieri di vino e un bicchiere di acqua naturale.

	COLAZIONE	Fuori pasto	PRANZO	Fuori pasto	CENA	Fuori pasto	ATTIVITÀ FISICA
LUNEDÌ			45 + 28<br>39, 1<br>49 + 49<br>52				☐ Nessuna<br>☐ Passeggiata<br>☐ Lavori domestici<br>☐ Palestra<br>☐ Altro (specificare)

Nel pomeriggio ha mangiata 1 mela e ha bevuto un caffè con 1 cucchiaino di zucchero

	COLAZIONE	Fuori pasto	PRANZO	Fuori pasto	CENA	Fuori pasto	ATTIVITÀ FISICA
LUNEDÌ				3<br>64 +63			☐ Nessuna<br>☐ Passeggiata<br>☐ Lavori domestici<br>☐ Palestra<br>☐ Altro (specificare)

A cena ha mangiato un piatto di minestrone senza pasta condito con olio extra vergine d' oliva crudo, una fetta di pane integrale, 1 pera e ha bevuto 2 bicchieri di acqua naturale.

	COLAZIONE	Fuori pasto	PRANZO	Fuori pasto	CENA	Fuori pasto	ATTIVITÀ FISICA
LUNEDÌ	30+64+63<br>3		45 + 28<br>39, 1<br>49 + 49<br>52	3<br>64 +63	Minestrone + 28<br>Pane integrale<br>3<br>52 + 52		☐ Nessuna<br>☒ Passeggiata<br>☐ Lavori domestici<br>☐ Palestra<br>☐ Altro (specificare)

Nell' ultima colonna, quella relativa all' attività fisica svolta durante la giornata, ha crocettato la passeggiata

COGNOME ______________________ NOME ______________________ CODICE ______________

MESE DI ______________________ Settimana dal ______________________ al ______________

	COLAZIONE	Fuori pasto	PRANZO	Fuori pasto	CENA	Fuori pasto	ATTIVITÀ FISICA
Lunedì							☐ Nessuna<br>☐ Passeggiata<br>☐ Lavori domestici<br>☐ Palestra<br>☐ Altro (specificare) ______
Martedì							☐ Nessuna<br>☐ Passeggiata<br>☐ Lavori domestici<br>☐ Palestra<br>☐ Altro (specificare) ______
Mercoledì							☐ Nessuna<br>☐ Passeggiata<br>☐ Lavori domestici<br>☐ Palestra<br>☐ Altro (specificare) ______
Giovedì							☐ Nessuna<br>☐ Passeggiata<br>☐ Lavori domestici<br>☐ Palestra<br>☐ Altro (specificare) ______
Venerdì							☐ Nessuna<br>☐ Passeggiata<br>☐ Lavori domestici<br>☐ Palestra<br>☐ Altro (specificare) ______
Sabato							☐ Nessuna<br>☐ Passeggiata<br>☐ Lavori domestici<br>☐ Palestra<br>☐ Altro (specificare) ______
Domenica							☐ Nessuna<br>☐ Passeggiata<br>☐ Lavori domestici<br>☐ Palestra<br>☐ Altro (specificare) ______

REPVBBLICA ITALIANA

Ministero
della Salute

ISTITUTO NAZIONALE di GASTROENTEROLOGIA
DE BELLIS
CASTELLANA GROTTE - ITALIA
ISTITUTO di RICOVERO e CURA a CARATTERE SCIENTIFICO

LABORATORI DI RICERCA
I.R.C.C.S. "Saverio De Bellis"
Castellana Grotte (BA)
tel. 080 4994650 / 651
