# Supplementary material for: Physical Activity and Low Glycemic Index Mediterranean Diet: Main and Modification Effects on NAFLD Score. Results from a Randomized Clinical Trial
Source: Nutrients. 2020 Dec 28;13(1):66. doi: 10.3390/nu13010066 (PMC7823843; doi:10.3390/nu13010066)
Supplement: Supplementary file 1 [file nutrients-13-00066-s001.zip › nutrients-1053716 - Supplementary S2 - Detailed information about LGIMD.pdf]

progetto NUTRI ATT

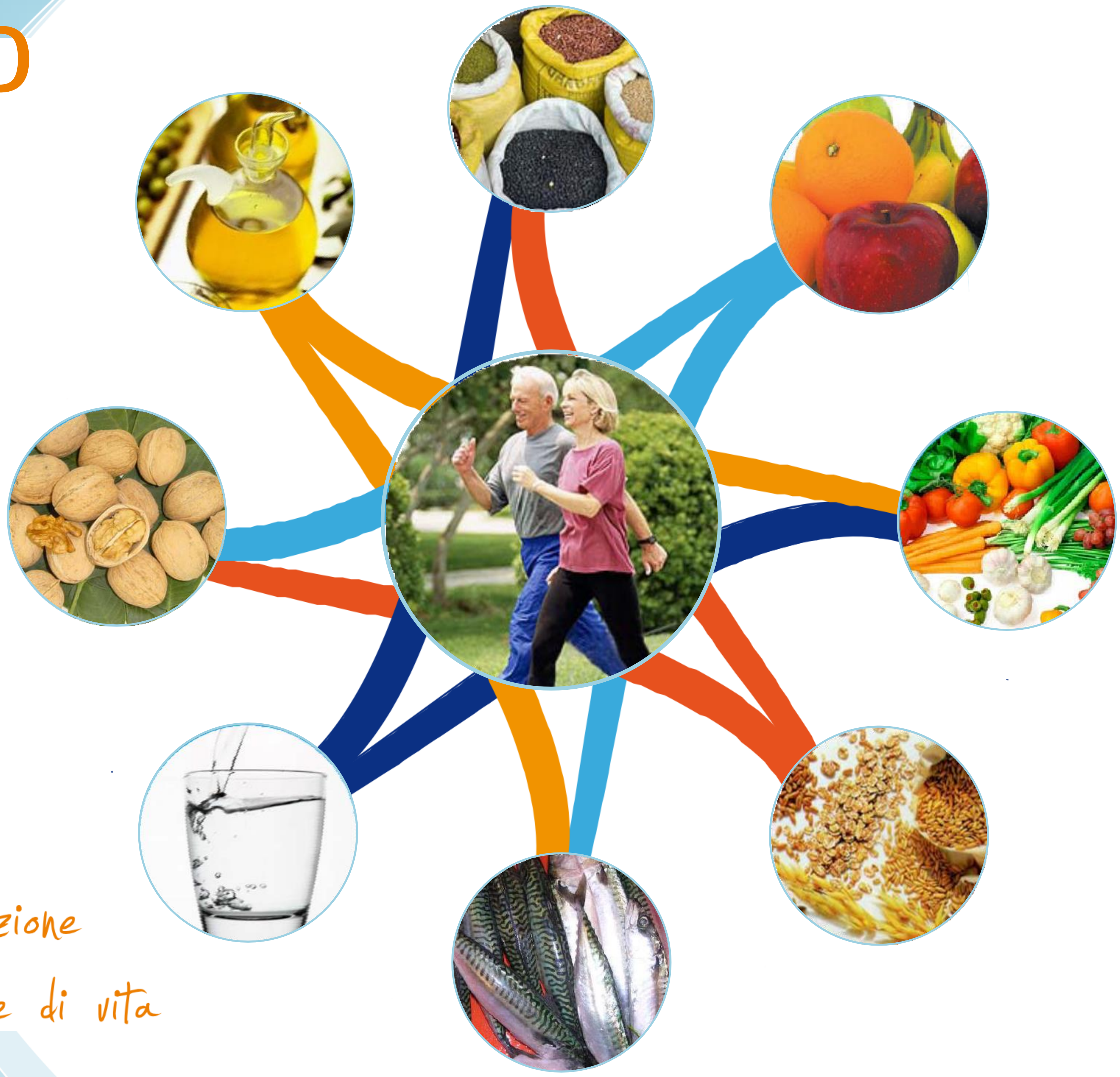

Una sana alimentazione

Un corretto stile di vita

PREMESSA

La salute è nelle tue mani, la costruisci ogni giorno quando vai al supermercato e scegli un cibo piuttosto che un altro, quando prepari un pranzo o una cena. In questi momenti fai scelte alimentari che influenzeranno profondamente la tua salute. Infatti una scorretta alimentazione determina la comparsa di una serie di fattori di rischio per le malattie cardiovascolari ed i tumori.

Una delle prime manifestazioni di questi fattori di rischio è rappresentato dalla Steatosi Epatica.
L'analisi dei tuoi dati fisiologici e di laboratorio, provenienti dallo studio Nutriep cui hai partecipato, ha evidenziato che hai la Steatosi Epatica (fegato grasso) e quindi sei a maggiore rischio di sviluppare una malattia cardiovascolare o un tumore, patologie che costituiscono la principale causa di morte nel mondo occidentale.
Il Laboratorio di Epidemiologia dell 'IRCCS "S. De Bellis" di Castellana Grotte ha attivato il progetto di Ricerca: "NutriAtt: Effetti della dieta Mediterranea a basso Indice Glicemico, di due programmi di attività fisica di diversà intensità e della loro interazione sulla steatosi Epatica di grado medio ed elevato e sulla composizione lipidica della membrana dei globuli rossi" .
Ti invitiamo, pertanto, a partecipare a questo studio che ci aiuterà a comprendere quale sia la dieta più efficace nel trattamento della Steatosi Epatica. Le indicazioni alimentari descritte nelle pagine seguenti sono quelle della Dieta Mediterranea a Basso Indice Glicemico, indicazioni che ti chiediamo di seguire. Pertanto ti preghiamo di registrare la tua dieta giornaliera su questo diario alimentare per tutto il periodo dello studio, per permetterci di valutare il tuo livello di aderenza alla Dieta che ti abbiamo consigliato.

ATTENTO AL SEMAFORO,

CONTROLLA LE TUE SCELTE ALIMENTARI

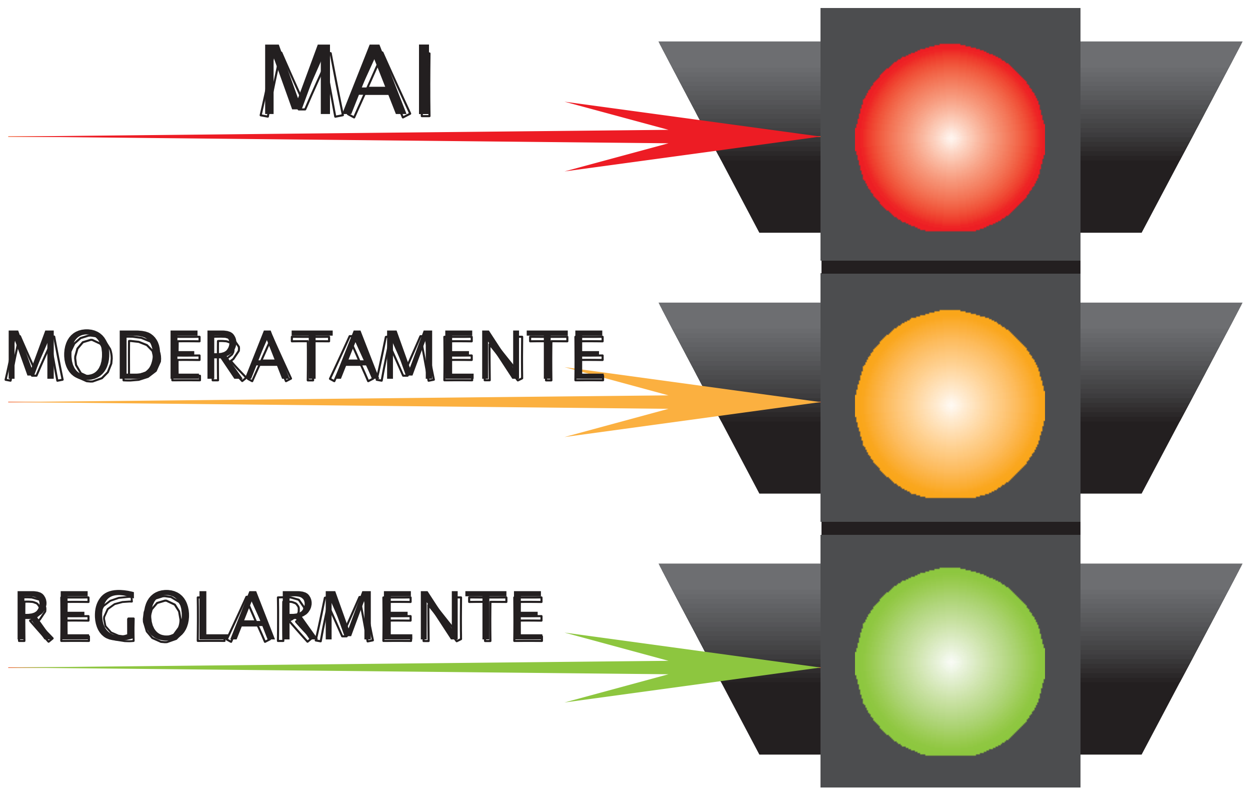

La scelta degli alimenti e la frequenza della loro assunzione è cosa fondamentale per impostare un corretto programma alimentare che, giorno per giorno, dovrebbe essere seguito da tutti per mantenere uno stato di salute soddisfacente.

Per orientare le scelte degli alimenti che meglio rappresentano la DIETA MEDITERRANEA A BASSO INDICE GLICEMICO, abbiamo contraddistinto con il colore VERDE i cibi da assumere regolarmente, con il GIALLO quelli da consumare con moderazione e con il ROSSO quelli da evitare.

REGOLARMENTE

1 VERDURA CRUDA (insalata verde, pomodori, cetrioli, sedano, carote, ravanelli, ecc.)<br>2 VERDURA COTTA A VAPORE O LESSATA (bietole, rape, cicorie, cavolfiori, broccoli, ecc.)	2 volte/dì
45 LEGUMI SECCHI (da soli) (lenticchie, ceci, fagioli, fave, soia)<br>46 LEGUMI FRESCHI (da soli) (piselli, fave, fagiolini)	3 volte/sett.
12- PASTA INTEGRALE CON LEGUMI<br>13- PASTA INTEGRALE CON VERDURA<br>18 RISO INTEGRALE CON LEGUMI<br>19 RISO INTEGRALE CON VERDURA	2 volte/sett.
42- PESCE AZZURRO (acciughe, sarde, sgombro)<br>40- MOLLUSCHI E CROSTACEI<br>43- MERLUZZO, PESCE SPADA, TONNO<br>66- SPIGOLA, ORATA, SALMONE (non di allevamento)<br>44- TONNO IN SCATOLA	3 volte/sett.
28- OLIO EXTRAVERGINE DI OLIVA (a crudo)	2 volte/dì.
5- FRUTTA FRESCA NON ZUCCHERINA (mele, pere, arance, pompelmo, kiwi, pesche, ecc.)<br>8- FRUTTA SECCA NON ZUCCHERINA (noci, mandorle)	3 volte/dì
65- CAFFE' (senza zucchero o con dolcificante artificiale)<br>52- ACQUA NATURALE	

MODERATAMENTE max

30 - LATTE E YOGURT	1 volta/dì
56 - BISCOTTI INTEGRALI	1 volta/dì (3 biscotti)
31 - FORMAGGI STAGIONATI (grana, parmigiano, pecorino, ecc.)<br>32 - LATTICINI (mozzarella, scamorza, ricotta)	2 volte/sett.
33 - UOVA	2 volte/sett. (un uovo)
34 - CARNE BIANCA (pollo, tacchino, coniglio)	2 volta/sett.
25 - PATATE (solo lesse)	1 volte/sett.
22 - PANE INTEGRALE	2 volte/dì (una fetta)
15 - PASTA INTEGRALE AL SUGO SEMPLICE	2 volta/sett. (max gr.80)
4 - FRUTTA FRESCA ZUCCHERINA (banane, cachi, uva)	1 volta/dì (max un pezzo)
62 - MIELE DENSO (puro)	1 volta/dì (1 cucchiaino)
49 - VINO	2 volte/dì (un bicchiere)

MAI

11- PASTA NON INTEGRALE
17- RISO NON INTEGRALE
21- PANE BIANCO
53- CRACKERS, SALATINI, GRISSINI, TARALLI, FOCACCE E CEREALI
47- PIZZA

35 CARNE ROSSA
(manzo, vitello, maiale, cavallo, ecc.)

36 CARNE IN SCATOLA
37 RAGU'
38 SALUMI, INSACCATI
(prosciutto crudo o cotto, bresaola, speck, salame, mortadella, ecc.)

41- PESCE DI ALLEVAMENTO
26- PATATE FRITTE O AL FORNO
67- FRITTURA IN GENERE
29- BURRO, MARGARINA, MAIONESE, PANNA
9- FRUTTA SCIROPPATA E CANDITA
7- FRUTTA SECCA ZUCCHERINA
(fichi secchi, datteri, prugne secche, uva sultanina)

63- ZUCCHERO

60 CARAMELLE E CIOCCOLATE
61 MARMELLATE
57 TORTE, DOLCI, PASTICCINI
58 CORNETTI, BRIOCHES, MERENDINE
54- BISCOTTI SECCHI, FETTE BISCOTTATE
55- FROLLINI, SAVOIARDI
59- GELATI E GRANITE
51- BEVANDE ALCOLICHE E SUPERALCOLICHE
(brandy, grappa, liquori, whisky)

50- BEVANDE NON ALCOLICHE GASSATE E NON
(aranciata, coca-cola, succo di frutta, ecc.)

48- BIRRA

IL TUO DIARIO ALIMENTARE

Istruzioni per la compilazione

- E’ fondamentale che il Diario venga compilato tutti i giorni dopo i pasti o appena possibile.
- Il Diario è costituito da una serie di fogli, uno per settimana. In ogni foglio settimanale, i giorni da lunedì a domenica sono suddivisi in colazione, pranzo, cena e fuori pasto.
- Registra tutti i cibi e tutte le bevande che consumi durante la giornata (compresi la domenica ed i giorni festivi).
- La registrazione consisterà nel riportare in corrispondenza del pasto il numero identificativo dell’alimento/bevanda o del gruppo di alimenti consumato che troverai nella lista che abbiamo allegato. Se non trovi nella nostra lista l’alimento che hai consumato, scrivi direttamente il suo nome nello spazio riservato al pasto corrispondente.

Esempio:

Descriviamo di seguito un ipotetico giorno del diario compilato dal Sig. Rossi.
Al termine di ciascun pasto il Sig. Rossi ha registrato ciò che ha mangiato e bevuto, per cui ha scritto nella casella del pasto specifico il numero corrispondente all’alimento consumato (consultando la lista degli alimenti).

A colazione ha bevuto una tazza di latte e caffè senza zucchero e ha mangiato una mela.

	COLAZIONE	Fuori pasto	PRANZO	Fuori pasto	CENA	Fuori pasto	ATTIVITÀ FISICA
LUNEDÌ	30+65<br>5						☐ Nessuna<br>☐ Passeggiata<br>☐ Lavori domestici<br>☐ Palestra<br>☐ Altro (specificare)<br>__________

A pranzo ha mangiato un piatto di lenticchie senza pasta condito con olio extra vergine d' oliva crudo, un merluzzo, un piatto di ortaggi crudi sconditi e ha bevuto due bicchieri di vino e un bicchiere di acqua naturale.

	COLAZIONE	Fuori pasto	PRANZO	Fuori pasto	CENA	Fuori pasto	ATTIVITÀ FISICA
LUNEDÌ			45 + 28<br>43 , 1<br>49 + 49<br>52				☐ Nessuna<br>☐ Passeggiata<br>☐ Lavori domestici<br>☐ Palestra<br>☐ Altro (specificare) ________

Nel pomeriggio ha mangiata 1 mela e ha bevuto un caffè senza zucchero

	COLAZIONE	Fuori pasto	PRANZO	Fuori pasto	CENA	Fuori pasto	ATTIVITÀ FISICA
LUNEDÌ				5<br>65			☐ Nessuna<br>☐ Passeggiata<br>☐ Lavori domestici<br>☐ Palestra<br>☐ Altro (specificare) ________

A cena ha mangiato un piatto di minestrone condito pasta condito con olio extra vergine d' oliva crudo, una fetta di pane integrale, 1 pera e ha bevuto 2 bicchieri di acqua naturale.

	COLAZIONE	Fuori pasto	PRANZO	Fuori pasto	CENA	Fuori pasto	ATTIVITÀ FISICA
LUNEDÌ	30+65<br>5		45 + 28<br>43, 1<br>49 + 49<br>52	5<br>65	Minestrone + 28<br>22<br>52 + 52		☐ Nessuna<br>☒ Passeggiata<br>☐ Lavori domestici<br>☐ Palestra<br>☐ Altro (specificare) ________

Nell' ultima colonna, quella relativa all' attività fisica svolta durante la giornata, ha crocettato la passeggiata

COGNOME _ MESE DI ___________

NOME____ CODICE ___________________________

Settimana dal____ al ___________________________

	COLAZIONE	Fuori pasto	PRANZO	Fuori pasto	CENA	Fuori pasto	ATTIVITÀ FISICA
Lunedì							☐ Nessuna<br>☐ Passeggiata<br>☐ Lavori domestici<br>☐ Palestra<br>☐ Altro (specificare) ___________
Martedì							☐ Nessuna<br>☐ Passeggiata<br>☐ Lavori domestici<br>☐ Palestra<br>☐ Altro (specificare) ___________
Mercoledì							☐ Nessuna<br>☐ Passeggiata<br>☐ Lavori domestici<br>☐ Palestra<br>☐ Altro (specificare) ___________
Giovedì							☐ Nessuna<br>☐ Passeggiata<br>☐ Lavori domestici<br>☐ Palestra<br>☐ Altro (specificare) ___________
Venerdì							☐ Nessuna<br>☐ Passeggiata<br>☐ Lavori domestici<br>☐ Palestra<br>☐ Altro (specificare) ___________
Sabato							☐ Nessuna<br>☐ Passeggiata<br>☐ Lavori domestici<br>☐ Palestra<br>☐ Altro (specificare) ___________
Domenica							☐ Nessuna<br>☐ Passeggiata<br>☐ Lavori domestici<br>☐ Palestra<br>☐ Altro (specificare) ___________

M inistero
della Salute

ISTITUTO NAZIONALE di GASTROENTEROLOGIA
DE BELLIS
CASTELLANA GROTTE • ITALIA
ISTITUTO di RICOVERO e CURA a CARATTERE SCIENTIFICO

LABORATORI DI RICERCA
I.R.C.C.S. “Saverio De Bellis”
Castellana Grotte (BA)
tel. 080 4994650 /651
